# 110-million-years-old fossil suggests early parasitism in shrimps

**DOI:** 10.1038/s41598-023-40554-2

**Published:** 2023-09-04

**Authors:** Daniel Lima, Damares R. Alencar, William Santana, Naiara C. Oliveira, Antônio Á. F. Saraiva, Gustavo R. Oliveira, Christopher B. Boyko, Allysson P. Pinheiro

**Affiliations:** 1 Museum of Paleontology Plácido Cidade Nuvens, Santana do Cariri, CE Brazil; 2 Department of Geology, Postgraduate Program in Geosciences (PPGEOC), Federal University of Pernambuco-UFPE, Avenida Prof. Moraes Rego, 1235, Cidade Universitária, Recife, PE 50670-901 Brazil; 3 Department of Biological Sciences, Regional University of Cariri-URCA, Rua Carolino Sucupira, s/n, Crato, CE 63100-000 Brazil; 4 Department of Biology, Federal Rural University of Pernambuco-UFRPE, Rua Dom Manuel de Medeiros, Recife, PE 52171-900 Brazil; 5 Department of Biology, Hofstra University, Hempstead, NY 11549 USA; 6 Division of Invertebrate Zoology, American Museum of Natural History, New York, NY 10024 USA

**Keywords:** Palaeontology, Zoology

## Abstract

Direct evidence of paleo-parasitism in crustaceans is very scarce. Epicaridean isopods are obligatory parasites of crustaceans, including decapods such as crabs, shrimps, and lobsters. Their interaction with hosts is known from fossils as far back as the Jurassic through deformations of the branchial cuticle on the hosts. Their small size and low fossilization potential, outside of those larvae that have been found in amber, makes understanding the group’s evolution challenging. Here, we report the oldest evidence of paleo-parasitism in marine shrimps and an imprint of a putative adult parasite that appears to be an epicaridean isopod. Our results suggest that the parasite–host interaction between epicaridean isopods and marine shrimps started at least 110 million years ago, and the Tethys Sea was a possible dispersal pathway for this lineage of parasites during the Jurassic and Cretaceous, as known for other marine organisms through most of the Mesozoic and Cenozoic. The oldest fossil records of bopyrid swellings associated with a large number of decapods from the Jurassic in Europe suggest that the Tethys region was a center of epicaridean distribution as a whole. Recent parasitic isopods found on dendrobranchiate shrimps are restricted to the Indo-Pacific and may represent a relict group of a lineage of parasites more widely distributed in the Mesozoic.

## Introduction

Isopod crustaceans—woodlice, sowbugs, and their relatives—are a diverse and successful group that evolved many kinds of lifestyles, including parasitism^1^. Direct evidence of paleo-parasitism by isopods dates back 168 million years, but the records are still scarce^1–4^.The symbiosis between decapod crustaceans (e.g., crabs, shrimps, hermit crabs, lobsters, and others) and epicaridean isopods (Bopyroidea and Cryptoniscoidea) is well-illustrated in the literature and dates back to the Late Jurassic^3,4^. Epicaridea is a monophyletic group^5^ comprising almost 900 species of obligate parasites of crustacean hosts, with most species belonging to the globally distributed Bopyroidea^6–9^.

Decapods have an extensive fossil record throughout the Phanerozoic, but evidence of decapods as hosts of parasites are scarce, direct fossils of the parasites are very rare^10,11^ and no adult epicaridean body remains are known. To date only some cryptoniscus larvae preserved in Mexico, France and Myanmar ambers have been described^12–15^. A common way to recognize paleo-parasitism in the absence of body fossils of parasites is to identify the traces of structures formed as a result of the interaction between parasite and host^8^. The presence and growth of some epicaridean ectoparasites causes swelling or deformation of the cuticle of their hosts, often in the branchial region of decapod crustaceans, which are relatively easy to recognize even in the fossil record^4,6,8^. The oldest undisputed fossil epicaridean swellings are known from the Jurassic (Oxfordian, Late Jurassic, 163–157.3 Ma) for hosts in Anomura and Brachyura^4,16^. These epicaridean-induced swellings in the branchial chambers of fossil decapod crustaceans have been given the formal ichnotaxon name of *Kanthyloma crusta*^4^.

The Lagerstätten from the Araripe Basin are well-known fossiliferous deposits noted for their rich paleozoological record that includes fishes^17^, dinosaurs^18^, turtles^19^, pterosaurs^20,21^, crocodilians^22^, and hundreds of invertebrate taxa such as echinoderms, arachnids, and crustaceans (including insects)^23–29^. Some Araripe fossils also show direct evidence of animal interactions involving the fish community, such as predator–prey relationships and cannibalism^30^, parasitism by copepods^31^, direct evidence of spinosaur consumption^32^, and insect-plant interactions, such as herbivory, galling, oviposition, and skeletonization^33^. This rich paleoenvironment yielded the parasite–host interaction between a putative epicaridean bopyrid parasite and a dendrobranchiate shrimp in the fossil record. After describing the epicaridean swelling, we discuss the perspective of this finding in terms of biogeography and early distribution of Bopyridae.

## Results

**Figure 1:**
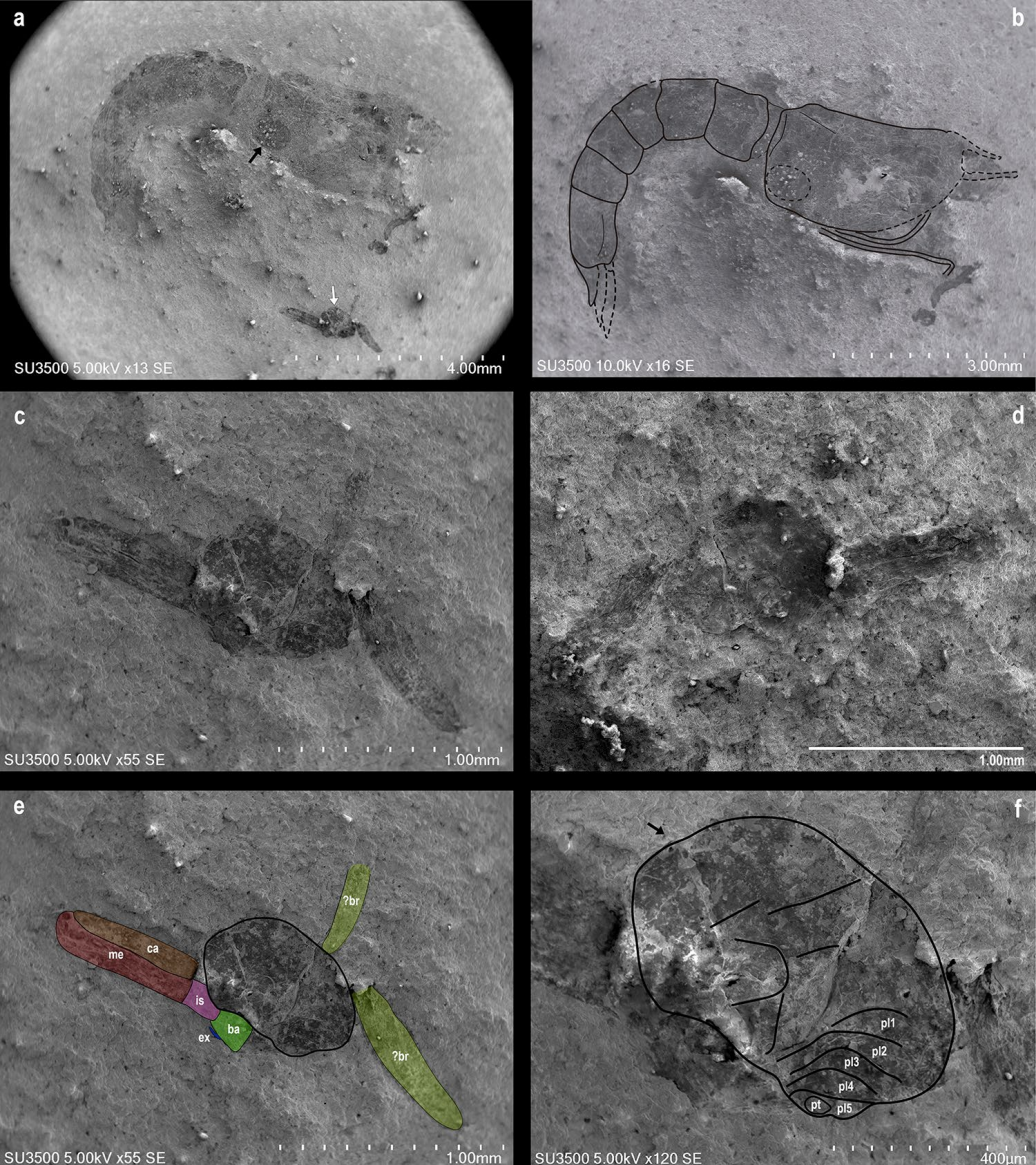
Parasite-host interaction. (**a**), Fossil dendrobranchiate shrimp, *Araripenaeus timidus* Pinheiro, Saraiva & Santana, 2014^34^ (lateral view) and the putative epicaridean isopod imprints. The black arrow shows the characteristic swelling in the shrimp branchial chamber indicating infestation by an epicaridean isopod. The white arrow indicates the unidentified fossil body imprint. (**b**), Shrimp line drawing. Dashed-lines show the non-preserved parts and the branchial chamber swelling. (**c**, **d**), Part and counter part of the unidentified fossil remains attached to the pereopods of *Araripenaeus timidus*. (**e**), Detail of the suboval fossil body attached to the shrimp’s pereopods. Pereopod segments: ?br, ?branchiae; ba, basis; ex, exopodite; is, ischium; me, merus; ca, carpus. Black line denotes the body outline. (**f**), Putative interpretation of the bopyrid outline, pleomeres 1–5 (pl1–pl5), and pleotelson (pt), arrow indicates anterior end of specimen.

The shrimp specimen (3.0 mm carapace length) reported here was identified as the dendrobranchiate *Araripenaeus timidus* Pinheiro, Saraiva & Santana, 2014^34^ (Decapoda, Dendrobranchiata) and is a laterally preserved imprint in a dark shale fragment (Fig. 1a,b) without distortion of its symmetry. The shrimp has a clear suboval convex swelling in the posterolateral carapace surface, just above the branchial chamber, which appears as a shallow hole in the impression (Fig. 1a,b). The swelling is longer than high at 0.7 mm in length, orientated on the long axis at an angle of 15° to the host thoracic main axis, a position very similar to what is observed in modern bopyrid–decapod interactions (see Supplementary Fig. S1a–e online).

About 2.3 mm, just below to the fossil shrimp, there is an unidentified suboval fossil imprint (0.7 mm long and 0.6 mm high) also preserved in the dark shale over disarticulated pereopods (Fig. 1c–f). Such pereopods may belong to the shrimp due to their shape and dimensions when compared to the preserved articulated shrimp pereopods (Fig. 1b). The suboval fossil does not seem to belong to any shrimp body or appendix remains and it has some segmentation along its lengt﻿h.

## Discussion

The swelling on the shrimp carapace is a strong indication of a parasite-host interaction between dendrobranchiates and epicarideans. Many authors have reported parasite–host interactions between epicarideans and fossil decapod crustaceans since the mid-nineteenth century^4^; all these reports are based on the swellings in the branchial region, formally assigned by Klompmaker et al. to the ichnotaxon *Kanthyloma crusta*, which is morphologically similar to the swellings induced by bopyrids in modern decapods^4,10^. The history of fossilized branchial swellings was reviewed by Klompmaker et al.^4,8^^.^

The general swelling outline and the mode of host infestation, residing within the branchial chamber of a decapod host, suggest that it may be attributed to the family Bopyridae (Epicaridea, Bopyroidea). The parasite–host interaction with a dendrobranchiate shrimp suggests a relation to the bopyrid subfamily Orbioninae, in which all members are exclusively branchial parasites of extant penaeoids. Living bopyrids of this subfamily comprise 39 species^35^, distributed only in the Indo-Pacific region^3,36^. This distribution pattern is very unusual, as their host group is considered ancient and well represented in all temperate and tropical waters^3,37^. However, the geographical distribution of Orbioninae indicates that they have evolved subsequent to the closing of the circumtropical Tethys Sea, no earlier than the Eocene^3,38^ but the first and, to date, only molecular study on Orbioninae phylogeny did not address estimations of divergence^39^.

Evidence of parasitism from the Early Cretaceous (Aptian) of the Araripe Basin suggests a much older origin for those bopyrids that have dendrobranchiate shrimps as their definitive hosts. Although Orbioninae is now restricted to the Indo-West Pacific, and this region is also probably the center of distribution for Bopyridae as a whole^3^, the oldest fossil records of bopyrids came from Europe (Late Jurassic). In addition, all the established records from the Jurassic and almost all from the Early Cretaceous also came from Europe^4^, reinforcing that the Tethys region had a key role in the early distribution of bopyrids.

**Figure 2:**
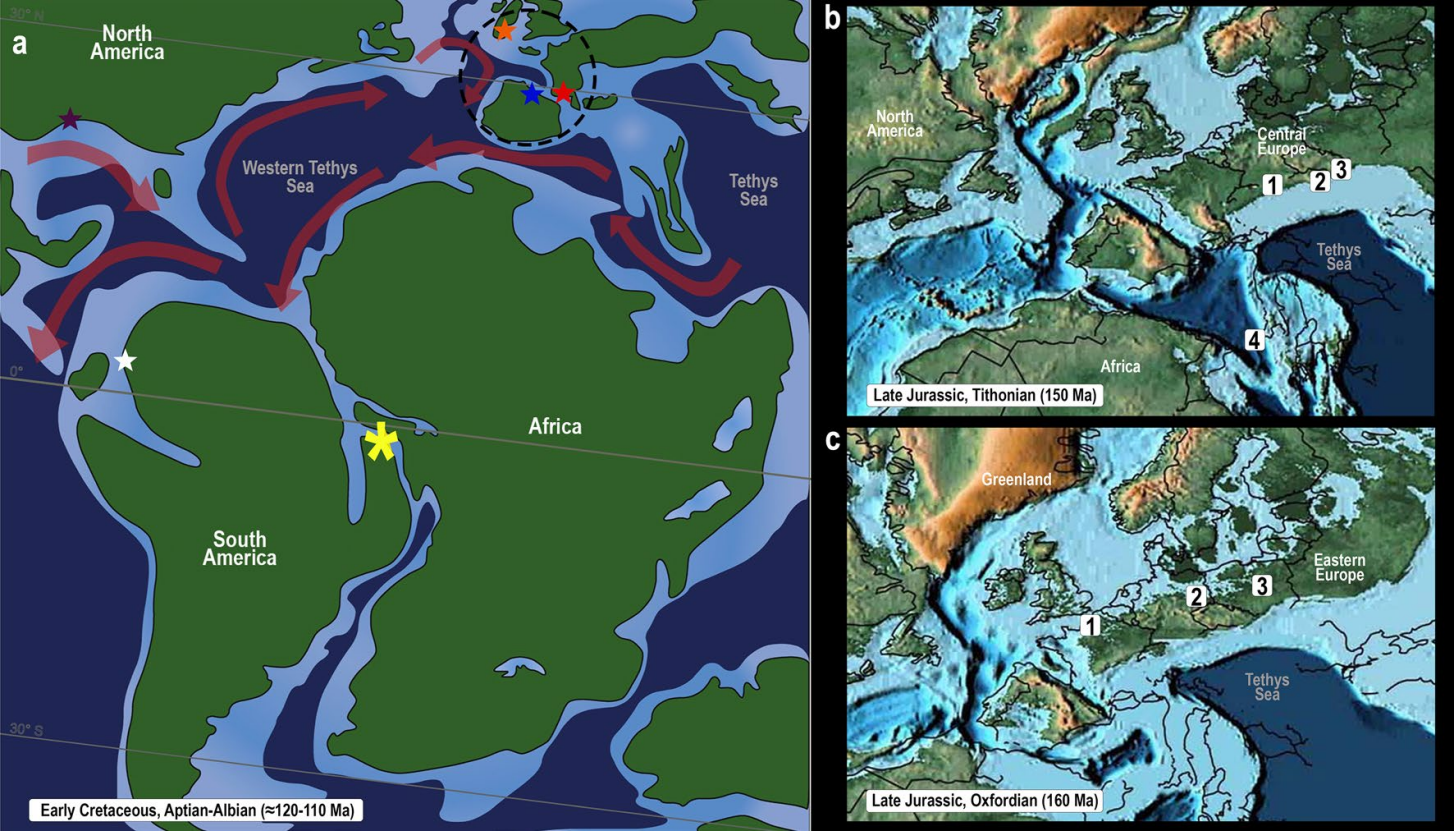
Paleomaps. (**a**), Early Cretaceous, Aptian-Albian (≈ 120–110 Ma). Colored stars indicate bopyrid occurrences in the Early Cretaceous based on records of the ichnotaxon *Kanthyloma crusta* after Klompmaker et al.^8^ Purple star, Aptian, United States; blue star, Albian, Spain; red star, Albian, France; orange star, Albian, England; white star, Aptian, Colombia; the yellow asterisk represents the bopyrid fossil imprint recorded in this study from the Araripe Basin, Aptian, Brazil. Dashed line denotes the putative center of irradiation of bopyrids in the Mesozoic. Red arrows represent the ocean paleocirculation during the Early Cretaceous. (**b**), Late Jurassic, Tithonian (150 Ma). 1, Austria; 2, Czech Republic; 3, Poland; 4, Italy. (**c**), Late Jurassic, Oxfordian (160 Ma). 1, France; 2, Germany; 3, Poland. (**a**), modified after Lúcio et al.^48^; (**b**) and (**c**), modified after Scotese^49^).

Evidence of parasite–host interaction between bopyrids and penaeoid shrimps from the Early Cretaceous suggests a much earlier interaction than the putative origin of orbionines, no later than the Eocene. If the present swelling was left by an orbionine, this subfamily may represent a Tethyan relict of a lineage widely distributed in the Mesozoic, although if it belongs to Bopyrinae, a subfamily of Recent caridean shrimp parasites, the pathway is less clear as members of that subfamily are broadly distributed worldwide today; it is also possible that it belongs to an extinct lineage of bopyrids. The Tethys was a well-known dispersal pathway for marine organisms through most of the Mesozoic and Cenozoic. A substantial number of taxa that originated in the Tethyan region dispersed east and west within the tropics, presumably via the narrow Atlantic^40–43^. The occurrence of the ichnotaxon *K. crusta* in the Mesozoic and Cenozoic from western Europe, southern North America, and northern South America^4^ suggests a Tethyan marine influence in the distribution of bopyrids (Fig. 2).

Alternatively, infestation in dendrobranchiate shrimps may have evolved more than once in Bopyridae, a phenomenon reported for other bopyrid lineages whose species parasitize several decapod higher taxa, such as Pseudioninae (with hosts in Brachyura, Anomura, Gebiidae, Axiidea, Astacidea, and Caridea) and Keponinae (hosts in Brachyura, Gebiidea, Axiidea, and Achelata)^7^.

Although the origin of Dendrobranchiata is supported by molecular phylogenetic studies that date it back to the early Silurian (437 Ma)^44^ and the group has the best fossil record among shrimp-like decapods (Dendrobranchiata, Caridea, Stenopodidea), with at least 79 known taxa^45,46^, there is no evidence of infestation by epicarideans in fossil dendrobranchiate shrimps to date. The only evidence of bopyrid parasitism of a shrimp-like species is recorded for *Axiopsis sampsonumae* Franţescu*,* 2014^47^, a mud shrimp (Axiidea) from the Lower Cretaceous of the United States; axiideans, however, are not closely related to dendrobranchiates.

The lower fossilization potential of shrimp-like species due to their generally thinner mineralized exoskeletons (compared to, e.g., brachyurans) may explain the fact that records are commonly found in deposits with a relatively advanced degree of fossil preservation^10,50,51^. For bopyrids on dendrobranchiates, the estuarine and oceanic ecological niches of hosts (that may have excluded most bopyrids to parasitize them) and the potential bopyrid preference for specific copepod intermediate hosts rather than the selection of definitive hosts may also explain the relative scarcity of records^36^.

Most recent species with evidence of epicaridean infestation are carideans and anomurans which comprise more than half of the known parasite-host interactions^3^. This evidence in part contrasts to what is seen in the fossil record where the oldest and most abundant records of bopyrid infestation are for brachyurans and galatheoids (Anomura), with the highest number of records from the Upper Jurassic^4,52^. The relative likelihood of preservation for hosts with thicker mineralized exoskeletons (crabs and squat lobsters) may explain this discrepancy.

Bopyrid parasites may have infested hosts in more recently diverged decapod infraorders, e.g., brachyurans and squat lobsters, first and later switched to other decapods such as dendrobranchiates^36^. However, the record of an epicarid parasitizing a dendrobranchiate shrimp in the Early Cretaceous reported here suggests that the switch may have happened earlier than predicted by the above hypothesis or, as seems more likely, the scarcity of the earliest diverged decapod infraorders in the early records of isopod infestations may be related to a bias caused by the reduced fossilization potential of shrimp-like decapods throughout the Phanerozoic.

Although it is not possible to state with certainty due to the absence of reliable diagnostic characters, the general outline and the segmentation observed in the unidentified suboval fossil appear to be an epicaridean isopod. Its basal region has a pattern of segmentation that resembles a slightly asymmetrical five-segmented pleon of an epicaridean bopyrid (Fig. 1c,e,f) and is most similar to species in genera of Bopyrinae; the host is preserved without distortion of its symmetry, thus the asymmetry of the parasite is not an artifact of preservation. On its right side there are two filamentous structures, less preserved than the pereopod on the left side (Fig. 1c; Supplementary Fig. S1f.) that resemble in shape and size the gills of extant dendrobranchiate shrimps (Supplementary Fig. S1b–d). However, the individual branchial lamellae cannot be seen and these may be also remains of another pereopod without the clear article segmentation.

While the putative parasitic isopod is small relative to most bopyrid species, specimens of *Bopyrina abbreviata* Richardson, 1904^53^ can be as small as 0.65 mm (immature females) to 1.27 mm (ovigerous females)^54^ (Supplementary Fig. S1g). Alternatively, the small size of both the swelling and parasitic isopod, may be indicative that the swelling was caused by a juvenile or immature female prior to causing the large swellings seen in mature bopyrid infestations. It is not possible to compare the putative bopyrid fossil with extant species in any detail, as the required characters (maxilliped, oostegite 1, pleopods) are not discernible in the fossil. Placement into a higher-level taxon is not possible as host choice suggests Orbioninae while overall body shape suggests Bopyrinae; other epicaridean groups show no affinities in either aspect. As noted previously, this fossil could represent an extinct lineage of bopyrids.

Generally, bopyrid isopods are unlikely to be preserved due to their low fossilization potential and relatively fast rate of decay^51^. However, the rapid mineralization and taphonomic processes in the Romualdo Formation Cretaceous Konservat-Lagerstätte led to exceptional cases of preservation, including soft and non-mineralized tissues, fully articulated specimens, and 3-D preservation in vertebrates^55,56^, and invertebrates (e.g., planktonic shrimps and brachyuran larvae^57^ and luciferid shrimp eyes^58^). The swelling left by the activity of a parasitic isopod and the putative epicaridean body imprint can be another case of exceptional preservation reported for the Araripe Basin but it is largely due to the chance dislodgement of the putative bopyrid from the host branchial chamber, a fact that we cannot explain given the unusual nature of this occurrence. Finally, we would like to emphasize that our current interpretation is based on the quality of the available material. Future studies may either support or contradict our interpretations and hypotheses patterns.

## Methods

### Locality and geological setting

The Araripe Basin is located between the states of Ceará, Pernambuco, and Piauí, in the Northeast of Brazil; its territorial extension is estimated at about 12,000 km^2^ ^59–62^. This basin is famous for fossils with an excellent degree of preservation and its abundance and variety of fossil groups, such as animals, plants, and trace fossils^63^. Geologically, this basin is composed of sequences bounded by regional unconformities. Five sequences are currently recognized, namely Paleozoic, Pre-Rift, Rift, Post-rift I, and Post-rift II, represented by the geological formations Cariri (Paleozoic), Brejo Santo, Missão Velha, Abaiara, Barbalha, Crato, Ipubi, Romualdo, Araripina, and Exu, from the Jurassic and Cretaceous periods^64^.

The lithostratigraphic unit of the Santana Group is the result of the Post-Rift I phase and is composed of the Barbalha, Crato, Ipubi, and Romualdo formations^60,65^. The Romualdo Formation is almost entirely Aptian in age (P-270 palynozone)^66^ and composed of fine sandstones, conglomerates, marls, concretions, limestones, and shales as lithological constituents^67^. U/Pb geochronology studies of fossil fish dentine yield a dating of 110.5 ± 7.4 million of year from Romualdo Formation^68^. This formation is well known for its paleobotanical and paleozoological records of excellent preservation, including fishes^17^, dinosaurs^18^, turtles^19^, pterosaurs^21^, crocodilians^22^, and hundreds of marine, brackish, and freshwater invertebrates^25^. The paleoenvironment is considered a low-energy coastal lagoon with periodic marine ingressions and regressions recorded^69^.

### Material

**Figure 3:**
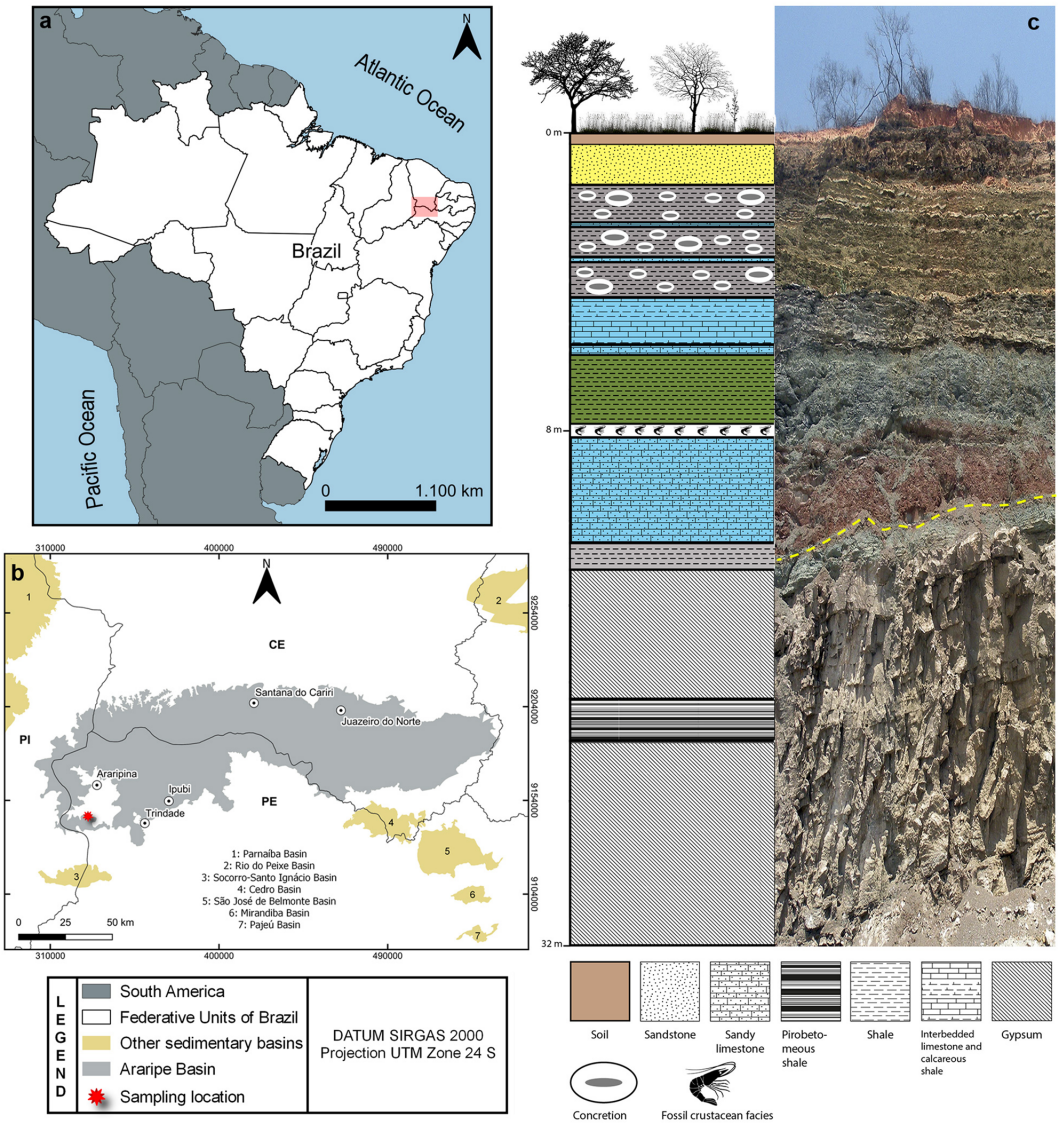
Sample location and stratigraphy. (**a**, **b**) Geographic position of the sampling site Trindade, Pernambuco, northeast Brazil, Araripe Basin. (**c**), Stratigraphic profile and scheme of the Romualdo Formation (Cretaceous – Aptian/Albian) where the specimen was collected; the yellow dashed line indicates the contact between Romualdo and Ipubi formations. Location maps (**a**, **b**) were created using QGIS software (version 3.16.16; https://www.qgis.org). Map figures (**a**) made with Natural Earth, and (**b**) was obtained from Brazilian Geological Service (CPRM, at https://geoportal.cprm.gov.br/geosgb/) powered by ESRI.

The specimen MPSC CR 5265 was collected in a paleontological expedition carried out in January 2016, from a single fossiliferous outcrop of the Romualdo Formation, located in the municipality of Trindade, state of Pernambuco, Northeast Brazil (07° 43′ 37.4″ S, 040° 32′ 26.8″ W) (Fig. 3). The single fossil specimen with part and counterpart was mechanically prepared. The specimen is deposited in the paleontological collection of the Museu de Paleontologia Plácido Cidades Nuvens (MPPCN) in Santana do Cariri, Ceará State, Brazil.

### Terminology

We essentially follow the morphological terminology used in previous studies of extant bopyrids^70^.

### Descriptions, drawings and photographs

A stereomicroscope Nikon SMZ800N equipped with a camera lucida and a Leica EZ4 W, with digital cameras attached were used for descriptions, drawings, and photographs. The software LAS EZ 3.4.0 [Build 272] was used to take the measurements, all in millimeters (mm). Line drawing and colored representations were made using Adobe Photoshop and Adobe Illustrator.

### Scanning electron microscopy

Micrographs were obtained in a SU3500 scanning electron microscope (Hitachi, Tokyo, Japan). The regions of interest were imaged using a SE detector, with accelerating voltages of 5, 8 and 10 kV. The fossil material was inserted into the microscope chamber without sample preparation, and the analyses were performed in high vacuum.

### Maps

The Early Cretaceous (Aptian) paleomap (Fig. 2a) illustrating the western Tethys Sea incursion in the northeast Brazilian basins was modified from ref.^48^. The Late Jurassic paleomaps (Fig. 2b,c) were modified from ref.^49^. Location maps 3a, b were created using QGIS software (version 3.16.16). Map figures from Fig. 3a were made with Natural Earth. Free vector and raster map data @naturelearthdata.com. Map figure from 3b was obtained from Brazilian Geological Service (CPRM, at https://geoportal.cprm.gov.br/geosgb/) powered by ESRI. Stratigraphic profile and scheme of the Romualdo Formation (Fig. 3c) (Cretaceous–Aptian/Albian) where the specimen was collected (modified from ref.^58^). Photos and illustrations were created and modified using Adobe Photoshop (v2022) and Adobe Illustrator (v2022).

### Supplementary Information

Supplementary Figure S1.

## Data Availability

All data generated or analyzed during this study are included in this published article [and its supplementary information files].
