# Supplementary material for: 110-million-years-old fossil suggests early parasitism in shrimps
Source: Sci Rep. 2023 Sep 4;13:14549. doi: 10.1038/s41598-023-40554-2 (PMC10477257; doi:10.1038/s41598-023-40554-2)
Supplement: Supplementary file 1 — Supplementary Figure S1. [file 41598_2023_40554_MOESM1_ESM.docx]

**Supplementary Information**


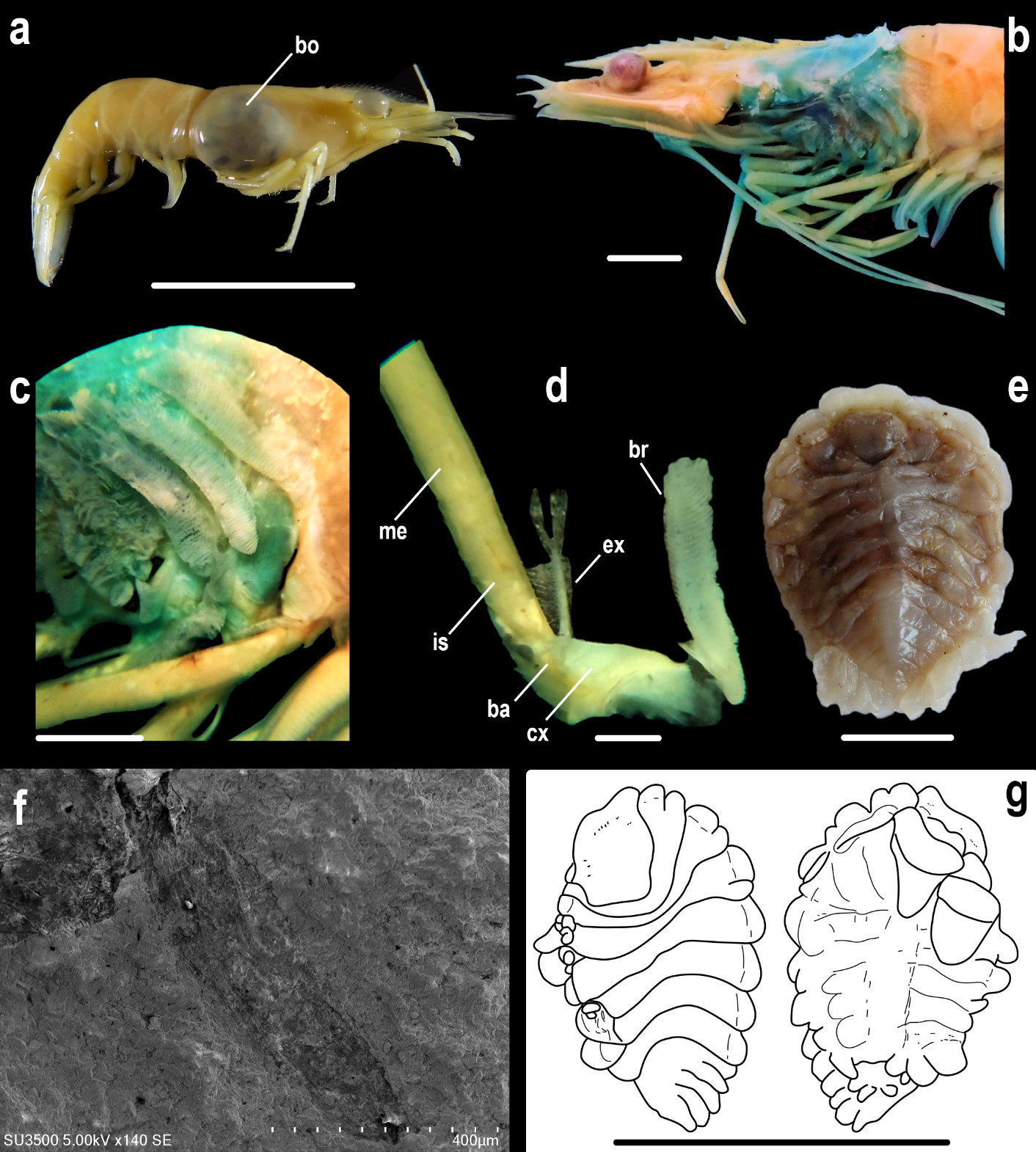


**Supplementary Fig. S1. Recent and fossil parasite-host interaction. a**, Extant caridean shrimp *Macrobrachium* *olfersii* (Wiegman, 1836)^71^ with bopyrid isopod infestation (bo) in the branchial chamber. **b**, Extant dendrobranchiate shrimp *Penaeus* sp. with exposed branchial chamber. The branchiae were stained with methylene blue. **c**, Branchial chamber detail of b. **d**, Proximal fourth pereopod detail. cx, coxae; ba, basis; ex, exopodite; is, ischium; me, merus; br, branchia. **e**, Extant orbionine bopyrid *Epipenaeon ingens* Nobili, 1906^72^ (extracted from ref.^73^). **f**, Branchia detail from Fig. 1e. **g**, Line drawing of an adult female of the bopyrine *Bopyrina abbreviata* Richardson, 1904^53^ (modified from ref.^74^) dorsal (right) and ventral (left) view. Scale bars: a, b, e= 10 mm; c= 5 mm; d= 2 mm; g= 1.5 mm.
